# Corticotrophin-Releasing Factor (CRF) and the Urocortins Are Potent Regulators of the Inflammatory Phenotype of Human and Mouse White Adipocytes and the Differentiation of Mouse 3T3L1 Pre-Adipocytes

**DOI:** 10.1371/journal.pone.0097060

**Published:** 2014-05-16

**Authors:** Eirini Dermitzaki, George Liapakis, Ariadne Androulidaki, Maria Venihaki, John Melissas, Christos Tsatsanis, Andrew N. Margioris

**Affiliations:** 1 Lab of Clinical Chemistry-Biochemistry, Department of Laboratory Medicine, School of Medicine, University of Crete, Heraklion, Crete, Greece; 2 Lab of Pharmacology, Department of Basic Sciences, School of Medicine, University of Crete, Heraklion, Crete, Greece; 3 Bariatric Unit, Department of Surgical Oncology, School of Medicine, University of Crete, Heraklion, Crete, Greece; University of California, Los Angeles, United States of America

## Abstract

Chronic activation of innate immunity takes place in obesity and initiated by the hypertrophic adipocytes which obtain a pro-inflammatory phenotype. The corticotrophin-releasing factor (CRF) family of neuropeptides and their receptors (CRF_1_ and CRF_2_) affect stress response and innate immunity. Adipose tissue expresses a complete CRF system. The aim of this study was to examine the role of CRF neuropeptides in the immune phenotype of adipocytes assessed by their expression of the toll-like receptor-4 (TLR4), the production of inflammatory cytokines IL-6, TNF-α and IL-1β, chemokines IL-8, monocyte attractant protein-1 (MCP-1) and of the adipokines adiponectin, resistin and leptin. Our data are as follows: (a) CRF, UCN2 and UCN3 are expressed in human white adipocytes as well as CRFR_1a_, CRFR_2a_ and CRFR_2b_ but not CRFR_2c_. 3T3L1 pre-adipocytes and differentiated adipocytes expressed both CRF_1_ and CRF_2_ receptors and UCN3, while UCN2 was detected only in differentiated adipocytes. CRF_2_ was up-regulated in mouse mature adipocytes. (b) CRF_1_ agonists suppressed media- and LPS-induced pre-adipocyte differentiation while CRF_2_ receptor agonists had no effect. (c) In mouse pre-adipocytes, CRF_2_ agonists suppressed TLR4 expression and the production of IL-6, CXCL1 and adiponectin while CRF_1_ agonists had no effect. (d) In mature mouse adipocytes LPS induced IL-6 and CXCL1 production and suppressed leptin. (e) In human visceral adipocytes LPS induced IL-6, TNF-α, IL-8, MCP-1 and leptin production and suppressed adiponectin and resistin. (f) In mouse mature adipocytes CRF_1_ and CRF_2_ agonists suppressed basal and LPS-induced production of inflammatory cytokines, TLR4 expression and adiponectin production, while in human visceral adipocytes CRF and UCN1 suppressed basal and LPS-induced IL-6, TNF-α, IL-8 and MCP-1 production. In conclusion, the effects of the activation of CRF_1_ and CRF_2_ may be significant in ameliorating the pro-inflammatory activity of adipocytes in obesity.

## Introduction

Corticotrophin releasing factor (CRF) is the principal regulator of the stress response. CRF and urocortins (UCNs) constitute a family of stress neuropeptides with high affinity to two known G-protein coupled receptors (type 1 CRF receptor, or CRF_1_ and type 2 CRF receptor, or CRF_2_). We and others have previously shown that CRF and its related peptides are fine regulators of the immune response exhibiting both pro- and anti-inflammatory effects via these receptors at the level of macrophages and mast cells [1]–[4].

Adipose tissue exerts systematic effects via a wide range of protein signals and factors, the adipokines, mainly involved in energy and immune homeostasis [5]. Human adipose tissue receives sympathetic innervation [6] and expresses the CRF family of peptides and its receptors [7]. Thus, the CRF stress neuropeptides reach adipose tissue either via the sympathetic innervation or via local production (paracrine effects). Evidence suggests that CRF affects several aspects of adipose tissue physiology. For example, it has been reported that CRF regulates adipocyte metabolism by down-regulating 11 beta-hydroxysteroid dehydrogenase, an enzyme converting inactive cortisone to the active compound cortisol [8].

Obesity is characterized by the development of generalized low grade chronic inflammation which is initiated within the micro-environment of adipose tissue where more and more monocytes and neutrophils are recruited by chemokines produced by the triglycerides-enriched adipocytes. White adipocytes and the monocytes/macrophages share the same immune machinery i.e. expression of the surface protein Toll-like receptor 4 (TLR4) (member of the TLR - Pattern Recognition Receptors family); its activation by fatty acids leads to the production of inflammatory cytokines and chemokines. Indeed, both types of cells respond to LPS via TLR4 producing inflammatory cytokines [9]. Furthermore, pre-adipocytes can differentiate to white mature adipocytes, and both types of adipocytes are sensitive to lipopolysaccharide (LPS) [10]–[12]. Pre-adipocytes exhibit phagocytic and anti-microbial activities towards microorganisms although to a lower degree compared to macrophages [13].

***The aim of this work*** was to study the expression of CRF system in both mouse pre-adipocytes and mouse white differentiated adipocytes and its involvement in adipocyte differentiation and immune phenotype. For this purpose, we examined the role of CRF peptides and their receptors in the differentiation of pre-adipocytes to mature white adipocytes and on the immune phenotype of both pre- and mature adipocytes in basal and upon activation by the TLR4 ligand LPS. We have found that both CRF receptors are expressed in mouse 3T3L1 pre-adipocytes and in their *in vitro* differentiation into mature, lipid-containing adipocytes as well as human visceral adipocytes. We have also found that the CRF_1_ and CRF_2_ agonists suppressed their differentiation and basal and LPS-induced inflammatory profile. CRF_2_ agonists mainly suppressed the inflammatory response while CRF_1_ agonists suppressed their differentiation.

## Materials and Methods

### Ethics Statement

The protocol was approved by the Ethics Committee of the University Hospital of Heraklion, Crete (No. 16901) and all participating subjects signed informed consents. The study was conducted as per the principles expressed in the Declaration of Helsinki.

### Reagents and Antibodies

Rat/human recombinant CRF was purchased from Tocris (Ellisville, MO), human UCN1, rat UCN1 (CRF_1_ and CRF_2_ agonists) and mouse UCN2 (CRF_2_ agonist) were obtained from Sigma. Cortagine (peptide CRF_1_ agonist), UCN3 (CRF_2_ agonist) and astressin-2B (CRF_2_ antagonist) were provided by Dr. J. Spiess (J. Burns School of Medicine, University of Hawaii, HI), whereas antalarmin (nonpeptide CRF_1_ antagonist) was provided by Dr. G. P. Chrousos (National Institutes of Health). DMEM, L-glutamine, fetal bovine serum (FBS), penicillin/streptomycin, and newborn bovine serum (NBC), were purchased from Invitrogen (Carlsbad, CA). Insulin, dexamethasone (DEX), sodium bicarbonate, 3-Isobutyl-1-Methyl-xanthine (IBMX), lipopolysaccharide (LPS) and Oil-Red-O were obtained from Sigma. Bradford Coomassie Brilliant Blue G-250 was obtained from Bio-Rad Laboratories, Inc. (Hercules, CA). All sterile tissue apparatus were obtained from Corning (Corning, NY). All other chemicals and reagents were obtained from Sigma, if not stated otherwise.

### 3T3L1 Pre-adipocytes Culture

3T3L1 pre-adipocytes were obtained from the American Type Culture Collection and cultured in a basal medium (DMEM, 4 mM L-Glutamine, 25 mM D-Glucose, 1 mM Sodium Pyruvate, 3.7 g/L NaHCO_3_, 50 units/ml penicillin, and 50 µg/ml streptomycin) supplemented with 10% NBS (growth medium) in a humidified atmosphere at 37 C. The medium was changed three times a week.

### 3T3L1 Differentiation

3T3L1 pre-adipocytes were plated in growth medium. The next day (Day 0) the media were changed to the differentiation media that composed of the basal medium supplemented with 10% FBS, 10 µg/ml insulin, 1 µM DEX and 0.5 mM IBMX. Two days later (Day 2) the media were changed to the basal medium supplemented with 10% FBS and 10 µg/ml insulin. The media were replaced every 2 days for a period of 8–14 days.

### Quantification of 3T3L1 Differentiation

Pre-adipocytes were plated at a density of 2,500 cells/96-well and forced to differentiate to mature adipocytes as mentioned above. In parallel, pre-adipocytes were exposed to CRF peptides plus/minus LPS during the differentiation process. The accumulation of lipid droplets in the cells’ surface (indicator of maturity of adipocytes) was evaluated by staining with Oil-Red-O. Briefly, cells were washed with PBS, incubated with 3.7% HCHO for 1 hr and then, incubated with Oil-Red-O solution for 45 min. Cells were washed very well to remove the excess of Oil-Red-O, visualized and photographed under an Olympus microscope with a Leica camera. For the quantification of cells’ differentiation, cells were dissolved in isopropanol and the optical density was read on a Dynatech Corp. Microelisa reader (Chantilly, VA) at 595 nm. The degree of the differentiation of the cells (accumulation of lipid droplets) was proportional to the optical density. Parallel wells incubated in growth or differentiating media supplemented with appropriate vehicles were assayed in every experiment to specify the net effect of CRF peptides and/or LPS in the differentiation process.

### Adipokines and Interleukins Measurements from 3T3L1

Pre-adipocytes were plated at a density of 200,000 cells/12-well. The next day, cells were exposed to CRF peptides plus/minus LPS at different time points and cell culture supernatants were collected and stored at −80 C until used for the determination of adipokine and interleukin concentration by ELISA.

Parallel experiments were performed in fully differentiated adipocytes. In brief, pre-adipocytes were cultured to 12-well plates at a density of 20,000 cells per well and forced to differentiate as mentioned above. Then, fully differentiated adipocytes were exposed to CRF peptides plus/minus LPS at different time points and cell culture supernatants were collected and stored at −80 C. Cell culture supernatants were also collected from adipocytes exposed to CRF peptides plus/minus LPS during the differentiation process.

Adiponectin was measured by MRP300 and Duoset (DY1119), while Leptin was measured by MOB00 and Duoset (DY498) purchased from R&D (R&D, NE). ELISA assays for IL-6 (DY406), CXCL1/KC (DY453), TNF-α (DY410), IL-1b (DY401) and IL-10 (DY417) were all purchased from R&D. For normalization of the measurements, cells were harvested and sonicated for quantification of total cellular proteins as previously described [14].

### Isolation and Culture of Human Primary White Adipocytes

Visceral adipose tissue biopsies were obtained from morbidly obese male subjects undergoing bariatric surgery. The adipose tissue was cut into small pieces and visible connective tissue and blood vessels were removed. The adipose tissue pieces were digested into D-PBS supplemented with 2% BSA and 0.5 mg/ml collagenase type I (Invitrogen) in a shaker-water bath at 37 C for 45 min. After centrifugation, the separation of mature adipocytes (floating white cellular layer) from stromalvascular fraction (pellet) was easily detectable. The corresponding white layer containing only mature adipocytes was collected and the number of cells was measured. Adipocytes were plated at a density of 800,000 cells/6-well in DMEM supplemented with 10% FBS and 1% penicillin/streptomycin for one day. The next day, cells were incubated in LPS (10 ng/ml) and/or 10^−8^ M CRF, UCN1 or appropriate vehicles at different time intervals. The cells and supernatants were collected and stored in −80 C for further analysis.

### Biochemical Measurements from Human Samples

The Human Adipocyte Magnetic Bead panel (Cat. # HADCYMAG-61K) from Millipore (Billerica, MA) was used for the simultaneous quantification of Adiponectin, HGF, IL-1β, IL-6, IL-8, Leptin, MCP-1, NGF, PAI-1 (total), Resistin and TNFα in the supernatants. The principle of the assay is based on the quantification of multiple bio-molecules concurrently employing fluorescent-coded magnetic beads (microspheres). The microspheres are incubated with the samples and then are allowed to pass rapidly through laser systems that distinguish the different sets of microspheres and the fluorescent dye on the reporter bio-molecules. The sensitivity of the assay for every bio-molecule is: 6.0 pg/ml Adiponectin, 2.6 pg/ml HGF, 0.5 pg/ml IL-1β, 0.4 pg/ml IL-6, 0.1 pg/ml IL-8, 4.7 pg/ml Leptin, 1.1 pg/ml MCP-1, 0.1 pg/ml NGF, 2.2 pg/ml PAI-1 (total), 2.6 pg/ml Resistin and 0.1 pg/ml TNFα. The intra-assays (%CV) for Adiponectin, HGF, NGF, MCP-1, TNFα and Resistin are both 2, for IL-6 and IL-8 are both 3, for Leptin is 16, for IL-1 β is 10 and PAI-1 (total) is 7. The inter-assays (%CV) for Adiponectin is 10, for HGF is 18, for NGF is 16, for MCP-1 is 9, for TNFα is 17, for Resistin is 8, for IL-6 is 15, for IL-8 is 12, for Leptin is 23, for IL-1 β is 12 and PAI-1 (total) is 17. IL6, IL8 and TNFα levels were also measured by chemiluminescence on an Immulite analyzer (Siemens).

### Isolation of Total RNA, PCR and RT-PCR

#### 3T3L1 cells

Pre-adipocytes and differentiated adipocytes were treated as described above and total cellular RNA was isolated using TRIzol Reagent (Invitrogen). Total cellular RNA from mouse heart and brain was also isolated and was used as positive controls for the expression of CRF peptides and their receptors. cDNA was prepared by reverse transcription (Thermoscript RT; Invitrogen) and amplified by PCR using the following primers pairs: for mouse β-actin, sense, 5′-TCA GAA GAA CTC CTA TGT GG-3′ and antisense, 5′-TCT CTT TGA TGT CAC GCA CG-3′, giving a 499-bp product; for mouse TLR4, sense, 5′-ACC AAT GCA TGG ATC AGA AA-3′ and antisense, 5′-GTC TCC ACA GCC ACC AGA TT-3′ resulting in a 295-bp product; for mouse CRF_1_, sense, 5′- GCC GCC TAC AAT TAC TTC CA -3′ and antisense, 5′- CGG AGT TTG GTC ATG AGG AT -3′, giving a 310-bp product; for mouse CRF_2_, sense, 5′- CTG GTG GCT GCT TTC CTG CTT TTC -3′ and antisense, 5′- ATG GGG GCC CTG GTA GAT GTA GTC C -3′, resulting in a 407-bp product; for mouse CRF, sense, 5′- AGC CCT TGA ATT TCT TGC A -3′ and antisense, 5′- AAC ACG CGG AAA AAG TTA -3′, resulting in a 202-bp product; for mouse UCN1, sense, 5′- ACT GGG CAG ACA CTC CGA TA -3′ and antisense, 5′- GGT AAG GGA AAG GGT CAA GG -3′, resulting in a 451-bp product; for mouse UCN2, sense, 5′- GGC CGC CGC TGA GAC TGA A -3′ and antisense, 5′- GGC CTG TGG ACC TTA GAT GGA CTT -3′, resulting in a 402-bp product; for mouse UCN3, sense, 5′- AGG TCC AAG GAC AAG CCT CT -3′ and antisense, 5′- TTT GAT CTG GAG GTG CGT TT -3′, resulting in a 196-bp product.

#### Human white adipocytes

Total cellular RNA was isolated from human white adipocytes using TRI Reagent (Sigma). Total cellular RNA from human placenta and hippocampus was also isolated and was used as positive controls for the expression of CRF peptides and their receptors. cDNA was prepared by reverse transcription (PrimeScript First Strand cDNA Synthesis Kit; Takara Bio, Inc.) and amplified by PCR using the following primers pairs: for human β-actin, sense, 5′-CCG GCC AGC CAG GTC CAG A-3′ and antisense, 5′-CAA GGC CAA CCG CGA GAA GAT G-3′, resulting in a 243-bp product; for human CRF, sense, 5′-CAC CCT CAG CCC TTG GAT TTC-3′ and antisense, 5′-GCC CTG GCC ATT TCC AAG AC-3′ resulting in a 413-bp product; for human UCN1, sense, 5′- CAG GCG AGC GGC CGC G-3′ and antisense, 5′- CTT GCC CAC CGA GTC GAA T-3′ resulting in a 146-bp product; for human UCN2, sense, 5′- GTG TCG GCC ACT GCT GAG CCT GAG AGA-3′ and antisense, 5′- ATC TGA TAT GAC CTG CAT GAC AGT GGC T-3′ resulting in a 195-bp product; for human UCN3, sense, 5′- TGC TGC TCC TGC TGC TGC TC-3′ and antisense, 5′- GTG TCC TGG CGT GGC TTT CCC-3′ resulting in a 310-bp product; for human CRFR_1a_, sense, 5′-GGC AGC TAG TGG TTC GGC C-3′ and antisense, 5′-TCG CAG GCA CCG GAT GCT C-3′ resulting in a 272-bp product; for human CRFR_2a_, sense, 5′-ATG GAC GCG GCA CTG CTC CA-3′ and antisense, 5′-CAC GGC CTC TCC ACG AGG G-3′ resulting in a 191-bp product; for human CRFR_2b_, sense, 5′-GGG GCT GGC CAG GGT GTG A-3′ and antisense, 5′-CAC GGC CTC TCC ACG AGG G-3′ resulting in a 342-bp product; for human CRFR_2c_, sense, 5′-CTG TGC TCA AGC AAT CTG CC-3′ and antisense, 5′-CAC GGC CTC TCC ACG AGG G-3′ resulting in a 300-bp product.

#### For PCR

A total of 1 µl of cDNA was used together with the primers shown above in a 20-µl reaction, using KAPA Taq PCR Kit (Kapa Biosystems, MA). Amplification was performed in a PCR apparatus for a maximum of 40 cycles as follows: 45 s at 94°C, followed either by 45 s at 55°C for mouse CRF, 45 s at 60°C for mouse β-actin, mouse CRF_1_, mouse CRF_2_, human CRF, human β-actin, 45 s at 57°C for mouse UCN1, mouse UCN3, human CRFR_1a_, human CRFR_2a_, human CRFR_2b_ and human CRFR_2c_, or 45 s at 64.5°C for mouse UCN2 and 45 s at 72°C. The amplification reactions were analyzed on 1.5% agarose gels.

#### For RT-PCR

A total of 1 µl of cDNA was used together with the primers for mouse TLR-4, mouse CRF_1_ and mouse CRF_2_ shown above in a 20-µl reaction, using SYBR green as a marker for DNA content, provided in the SYBR Green PCR Master Mix (Applied Biosystems). Amplification was performed in an ABI PRISM 7000 Real-Time PCR apparatus for a maximum of 40 cycles as follows: 40 s at 94°C, 40 s at 53°C (for TLR-4) or 30 s at 60°C (for CRF_1_ and CRF_2_), 1 min at 72°C. No by-products were present in the reaction as indicated by the dissociation pattern provided at the end of the reaction and by agarose gel electrophoresis (data not shown). The amplification efficiency of the products was the same as the one of β-actin as indicated by the standard curves of amplification, allowing us to use the formula: fold difference = 2^−(ΔCtA − ΔCtB)^, where Ct is the cycle threshold. Reactions were performed in triplicate to allow for statistical evaluation. Each experiment was repeated three times.

### FACS Analysis

Pre-adipocytes and differentiated adipocytes were stained for TLR4 as previously described [1]. In brief, at the end of the incubation periods cells were washed twice with PBS containing 1% BSA. Then, anti-mouse TLR4-PE-conjugated Ab (clone MTS510; e-Bioscience) was added, and cells were incubated at 4 C for 20 min. Cells were washed twice with PBS containing 1% BSA and analyzed on a flow cytometer (FACSCalibur; BD Biosciences).

### Binding Assays

3T3L1 pre-adipocytes or differentiated adipocytes were washed with PBS, at room temperature, treated with PBS containing 2 mM EDTA (PBS/EDTA), and then dissociated in PBS/EDTA. Membrane suspensions were prepared as described previously [15] and ^125^I-Tyr^0^-sauvagine (PerkinElmer, Waltham, USA) binding studies were performed as previously described [15]. Aliquots of membrane suspension (50 ml) were incubated with 350–400 pM ^125^I-Tyr^0^-sauvagine with or without 500 nM Astressin-2B and/or 1000 nM antalarmin in a final volume of 0.1 ml. The mixtures were incubated at 20–21 C for 2 h and then filtered through glass fiber filters (934AH; Whatman) presoaked for 1 h in 0.3% polyethylenimine at 4 C. The filters were washed 3 times with 0.5 ml of ice-cold PBS, pH 7.1, containing 0.01% Triton X-100 and assessed for radioactivity in a gamma counter (LKB Wallac 1275 minigamma, 80% efficiency).

### Statistical Analysis

For the statistical evaluation of our data, we used ANOVA, *post hoc* comparison of means followed by two multiple comparison tests: Fisher’s least significance difference and the Newman-Keuls test. For data expressed as percent changes, compared with control values, we used the nonparametric Kruskal-Wallis test for several independent samples.

## Results

### Characterization of Pre- and Mature 3T3L1 Adipocytes

3T3L1 pre-adipocytes cultured in standard growing conditions exhibited a rapidly growing fibroblast-like phenotype. As expected, the cells were not accumulating lipids as documented by negative staining with Oil-Red-O. In contrast, the fully differentiated adipocytes exhibited a much reduced proliferation rate and their phenotype was characterized by enlarged and rounded cytoplasm abundant with lipid droplets. Lipid droplet formation was easily observed by staining with Oil-Red-O (data not shown). Leptin secretion was undetectable in pre-adipocytes while it became abundant upon differentiation to mature adipocytes. Pre-adipocytes as well as mature adipocytes secreted adiponectin, IL-6 and CXCL1, while IL-1b, TNF-α and IL-10 were undetectable in both populations. Interestingly, basal adiponectin secretion was higher, while the basal CXCL1 was lower in the fully differentiated adipocytes compared to pre-adipocytes. The basal levels of IL-6 secretion were comparable in both populations (data not shown).

### Expression of CRF Receptors and Related-peptides in 3T3L1 Pre-adipocytes, Differentiated Adipocytes and Human Primary Visceral White Adipocytes

**Figure 1:**
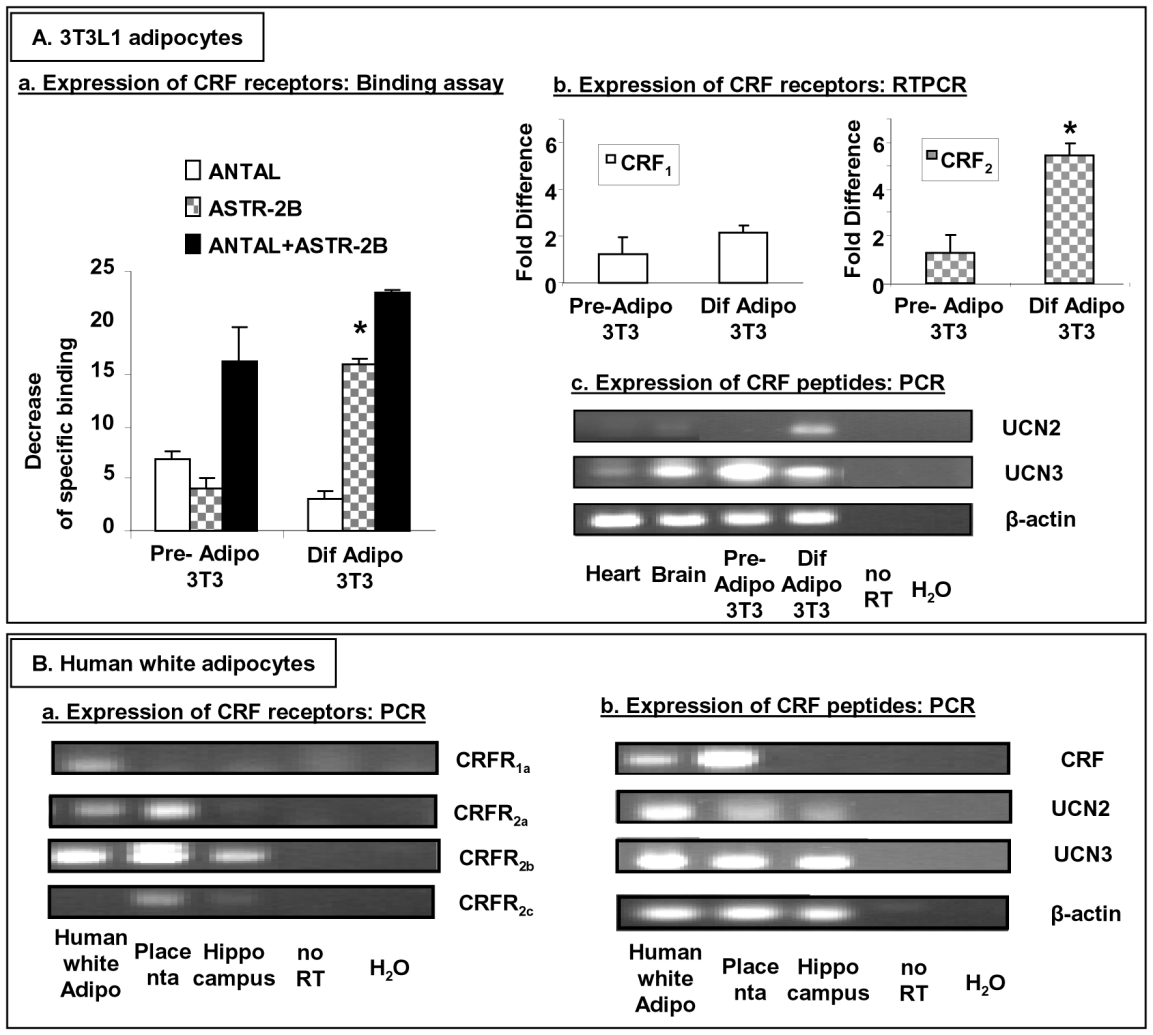
Expression of CRF receptors and peptides in 3T3L1 pre-adipocytes, differentiated adipocytes and human primary white adipocytes. Panel A, (a), Cells were exposed to antalarmin (a CRF_1_ receptor antagonist) and/or astressin-2B (a CRF_2_ receptor antagonist) and the decrease of specific binding activity was measured. Data are expressed as the decrease of specific binding (mean±SE, n = 4 of two independent experiments) and *p<0.05 denotes significant statistical difference between the levels of CRF_2_ expressed in pre-adipocytes compared to the levels of CRF_2_ expressed in differentiated adipocytes; (b), The mRNA levels of CRF_1_ and CRF_2_ was measured in 3T3L1 pre-adipocytes and differentiated adipocytes by RT-PCR. Data are expressed as the fold difference of the mRNA levels of CRF_1_ or CRF_2_ expressed in pre-adipocytes (mean±SE, n = 4 of two independent experiments) and *p<0.05 denotes significant statistical difference between the levels of CRF_2_ expressed in differentiated adipocytes compared to the levels of CRF_2_ expressed in pre-adipocytes; (c), The mRNA levels of UCN2 and UCN3 were measured by PCR in mouse samples. Panel B, The mRNA levels of CRF receptor isoforms (column a) and CRF peptides (column b) were measured in human samples. Beta-Actin was used for control.

The presence of CRF_1_ and CRF_2_ receptors was examined in pre-adipocytes and in fully differentiated adipocytes. For this purpose, we determined the ability of antalarmin (a CRF_1_ receptor antagonist) and/or astressin-2B (a CRF_2_ receptor antagonist) to decrease ^125^I-Tyr^0^-sauvagine binding to membrane preparations of pre-adipocytes and differentiated adipocytes. The results are depicted in **Figure 1** **, panel A, a**. Antalarmin and/or astressin-2B decreased radioligand binding to pre-adipocytes and differentiated adipocytes, suggesting that both CRF_1_ and CRF_2_ receptors are expressed in both types of adipocytes. Differentiation of adipocytes did not significantly affect the expression of CRF_1_ receptor. In marked contrast, the expression of CRF_2_ receptor in differentiated adipocytes was significantly higher compared to parallel pre-adipocytes. These results are in accordance with our data from RT-PCR. Indeed, the mRNA levels of CRF_1_ were comparable in pre-adipocytes and differentiated adipocytes, while the mRNA levels of CRF_2_ were five times higher in differentiated adipocytes compared to the mRNA levels of CRF_2_ in pre-adipocytes **Figure 1** **panel A, b**. We also used mouse heart and mouse brain for controls. It was shown that the mRNA levels of CRF_1_ were abundantly expressed in mouse brain and in a lesser extent in mouse heart. In contrast, the mRNA levels of CRF_2_ were detected in large quantities in the heart and in a lesser extend in brain (data not shown).

The mRNA of UCN2 was expressed in differentiated adipocytes but not in pre-adipocytes, while the UCN3 mRNA was expressed in pre-adipocytes, differentiated adipocytes and brain (**Fig. 1** **, panel Ac**). The mRNA levels of CRF or UCN1 were detectable neither in pre-adipocytes nor in differentiated adipocytes (data not shown).

The presence of CRF_1_ and CRF_2_ receptor subtypes as well as of CRF, UCN1, UCN2 and UCN3 was examined in human white adipocytes by PCR (**Figure 1B**). Human placenta and hippocampus were used for positive controls. CRF was expressed both in human white adipocytes and placenta. UCN2 and UCN3 were markedly expressed in human white adipocytes. UCN3 was also expressed in placenta and hippocampus. CRFR_1a_, CRFR_2a_, CRFR_2b_ but not CRFR_2c_ was expressed in human white adipocytes. Human placenta expressed CRFR_2a_, CRFR_2b_ and CRFR_2c_, while human hippocampus expressed CRFR_2b_ and weakly CRFR_2c_.

### Effect of CRF_1_ and CRF_2_ Agonists on the Differentiation Rate of 3T3L1 Pre-adipocytes to Mature Adipocytes

**Figure 2:**
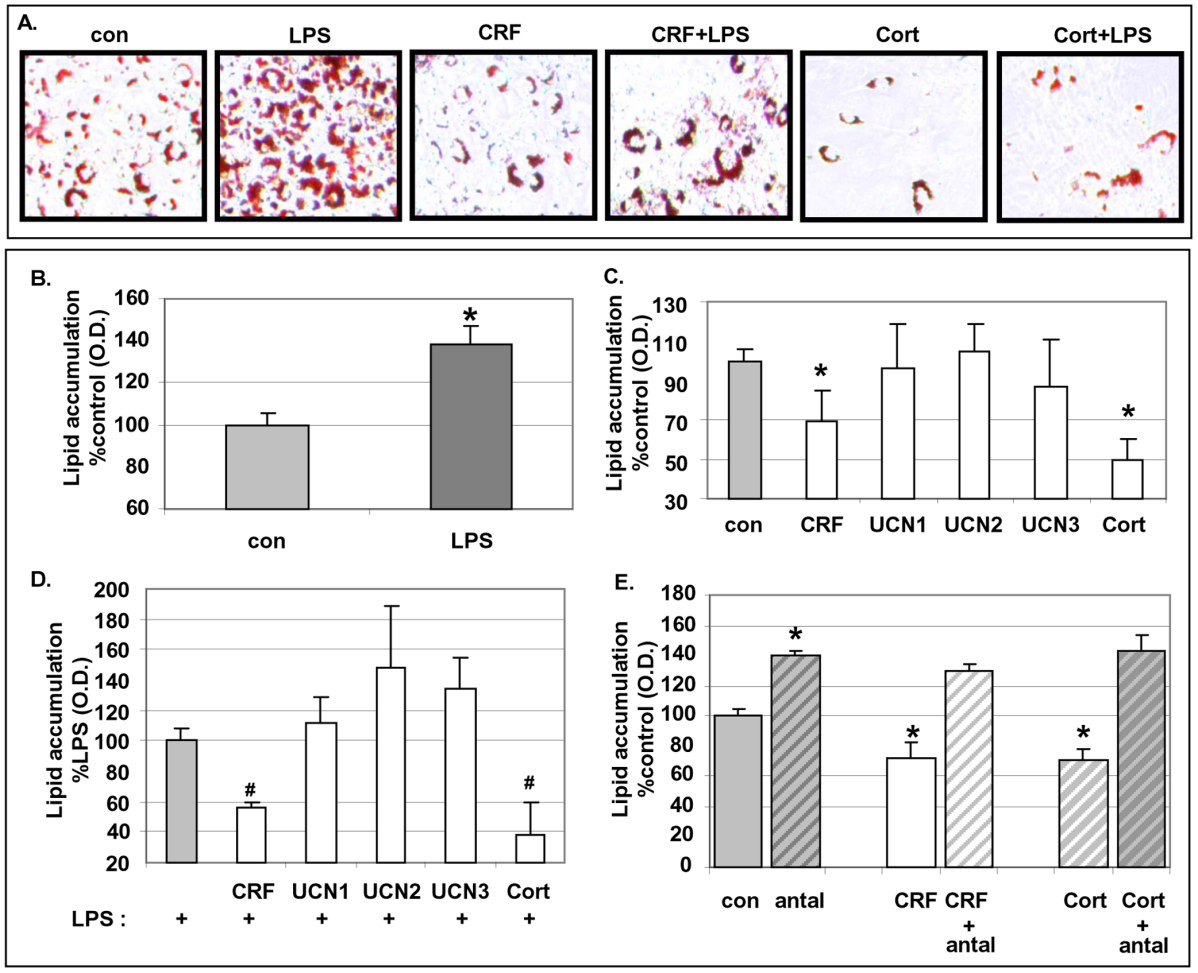
Effect of CRF agonists on the differentiation process of 3T3L1 cells. Pre-adipocytes were exposed to CRF peptides and/or LPS during the differentiation period and stained with Oil-Red-O. Panel A, The accumulation of lipid droplets were visualized under a microscope; Panels B, C and D, The degree of differentiation was measured as the intensity of the absorbance read at 595 nm. Panel E, The accumulation of lipid droplets of cells pre-exposed to antalarmin (10^−6^ M) and to CRF or Cortagine at 10^−8^ M was measured as the intensity of the absorbance read at 595 nm. Data are expressed as percentage change compared with control values (Panels B, C, E) or compared with LPS (Panel D) (mean±SE, n = 8 of three independent experiments). *p<0.05 depicts the statistical significant difference from cells exposed only to vehicles, while ^#^p<0.05 depicts the statistical significant difference from cells exposed to LPS alone.

Pre-adipocytes were induced to differentiate to mature white adipocytes under the standard differentiating conditions [16]. During the differentiation period, pre-adipocytes were exposed to CRF peptides at 10^−8 ^M in the presence or absence of LPS (10 ng/ml). Differentiation was confirmed by the accumulation of lipid droplets by Oil-Red-O staining (**Fig. 2A**) and quantified by optical densitometry (**Fig. 2B–E**). Exposure of pre-adipocytes to LPS during the differentiation period accelerated the process by 138±9% (mean±SE%, *p<0.05*) compared to parallel controls (100±6%) (**Fig. 2B**). Exposure of adipocytes to CRF or Cortagine (CRF_1_ agonists) during their differentiation period decelerated the process to 69±15% (*p<0.05*) or 50±10% (*p<0.05*), respectively compared to parallel controls (100±6%) (**Fig. 2C**). In addition, the presence of CRF or Cortagine significantly suppressed LPS-induced differentiation of adipocytes to 55±3% (*p<0.05*) and 38±20% (*p<0.05*), respectively compared to LPS-treated parallel adipocytes (**Fig. 2D**). Exposure of cells to CRF_2_ agonists during the differentiation process had no effect on either their basal differentiation rate or the LPS-induced differentiation. Pre-treatment of cell with antalarmin (CRF_1_ antagonist) reversed the effect of CRF or Cortagine on the accumulation of lipid droplets in adipocytes during differentiation (**Fig. 2E**).

### Effect of CRF_1_ and CRF_2_ Agonists and Antagonists on the Production of Inflammatory Cytokines during the Differentiation of 3T3L1 Pre-adipocytes to Mature Adipocytes

In this set of experiments we measured the production of interleukins IL-6, CXCL1 and of the adipokines adiponectin and leptin from adipocytes exposed to the CRF_1_ ligand Cortagine in the presence or absence of LPS during their differentiation period. Exposure to LPS during the differentiation period provoked a spectacular increase in IL-6 and CXCL1 production accompanied with a minor suppression of adiponectin while leptin production was unaffected compared to cells exposed only to the differentiation medium (**Fig. S1**). Exposure of adipocytes to Cortagine during the differentiation period suppressed the production of IL-6, CXCL1, adiponectin and leptin when compared to cells exposed only to the differentiation medium (**Fig. S1**).

**Figure 3:**
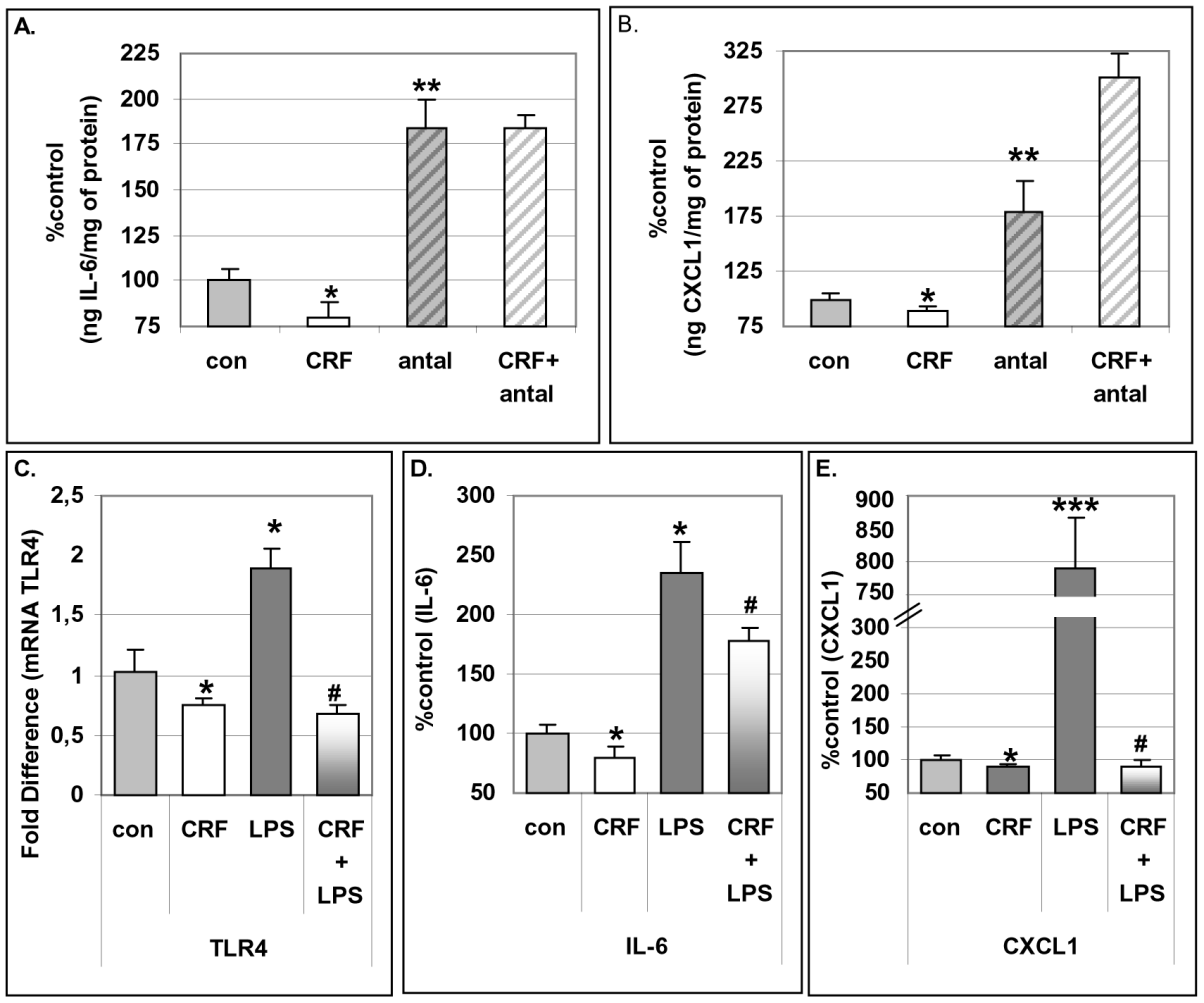
Effect of CRF on TLR4 and interleukins during differentiation of 3T3L1. Pre-adipocytes were cultured in differentiating media supplemented with CRF at 10^−8^M, pre-treated with antalarmin at 10^−6^ M and the production of IL-6 and CXCL1 was measured by ELISA (Panels A, B). Pre-adipocytes were cultured in differentiating media supplemented with CRF at 10^−8 ^M plus/minus LPS (10 ng/ml) and the expression of TLR4 was measured by RT-PCR (Panel C) and the production of IL-6 and CXCL1 (Panels D, E) was measured by ELISA. Data are expressed as percentage change compared with control values (mean±SE, n = 10 of five independent experiments). *p<0.05, **p<0.01, ***p<0.001 depict the statistical significant difference from cells exposed only to vehicles, while ^#^p<0.05 depicts the statistical significant difference from cells exposed to LPS alone.

CRF was less potent than Cortagine in suppressing IL-6 and CXCL1 production during the differentiation period, but it was still effective. Indeed, exposure of adipocytes to CRF during the differentiation period suppressed the production of IL-6 and CXCL1 when compared to cells exposed only to the differentiation medium (**Fig. 3A, B**). Antalarmin, a CRF_1_ antagonist, reversed the effect of CRF on either IL-6 or CXCL1 production (**Fig. 3A, B**), while Astressin-2B (CRF_2_ antagonist) was ineffective (data not shown).

### Effect of CRF_1_ and CRF_2_ Agonists on the Expression of TLR4 Receptor during the Differentiation of 3T3L1 Pre-adipocytes to Mature Adipocytes

Exposure of adipocytes to LPS during their differentiation period induced the expression of TLR4 (mRNA levels) (**Fig. 3C**) in parallel to the augmentation of IL-6 production (**Fig. 3D**) and CXCL1 production (**Fig. 3E**). Exposure of adipocytes to CRF during differentiation suppressed both basal and LPS-induced TLR4 mRNA expression as well as IL-6 and CXCL1 production (**Fig. 3C–E**).

### Effect of the CRF_2_ Agonists on Pre-adipocytes: Production of Interleukins and Expression of TLR4

**Figure 4:**
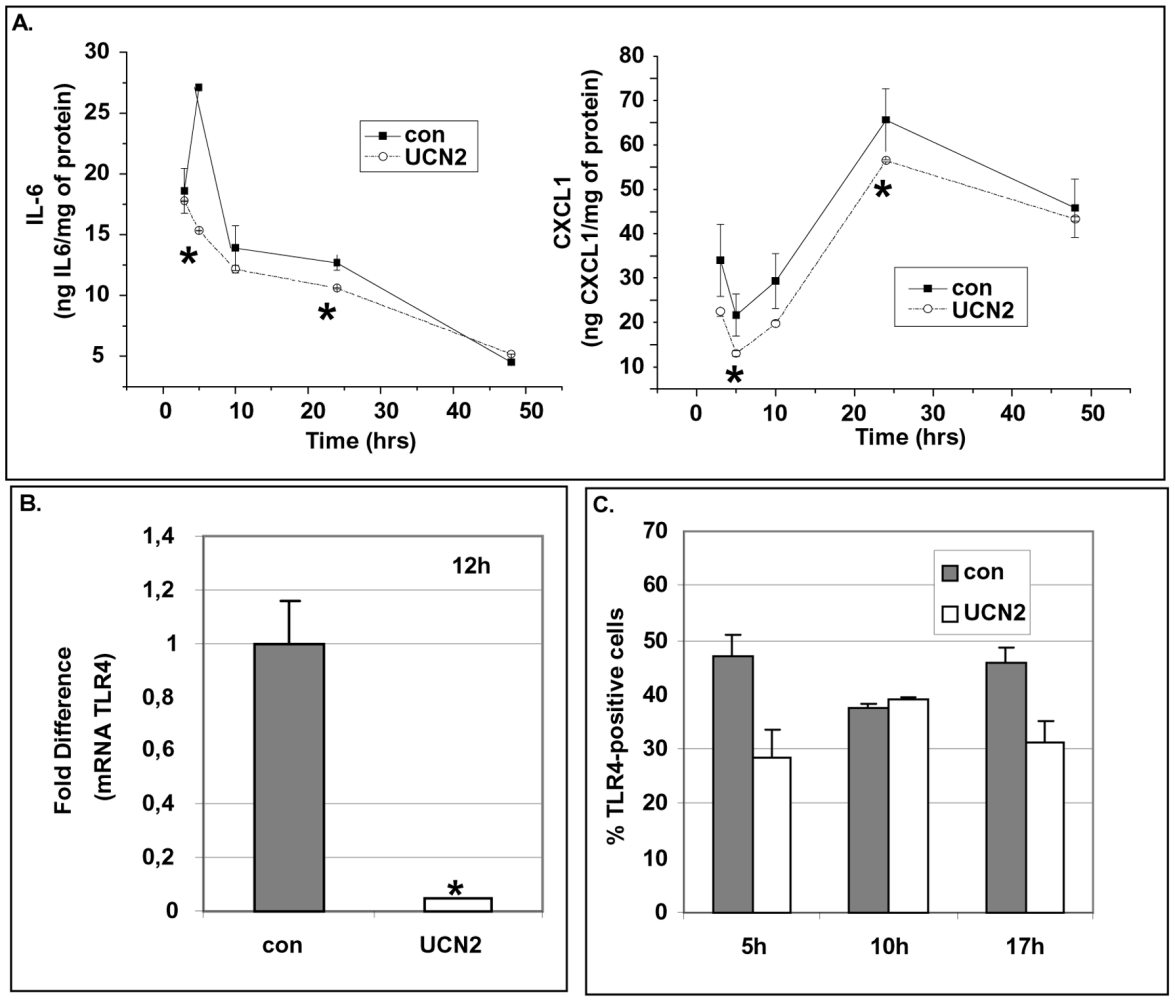
UCN2 affects the production of interleukins and the expression of TLR4 by pre-adipocytes. Pre-adipocytes were exposed to UCN2 at 10^−8^M and the production of interleukins was measured by ELISA (Panel A), the TLR4 mRNA levels were measured by RT-PCR (Panel B) and TLR4 protein levels were measured by FACS analysis (Panel C) at several time intervals. Data are expressed as mean±SE, n = 6 of three independent experiments. *p<0.05 depicts the statistical significant difference from cells exposed only to vehicles.

Pre-adipocytes were exposed to UCN2 (10^−8^M) and the production of IL-6 and CXCL1 was measured at different time intervals. UCN2 transiently suppressed basal production of IL-6 and CXCL1 by pre-adipocytes (**Fig. 4A**). UCN2 also suppressed the expression of TLR4 (mRNA levels) at 12 h (**Fig. 4B**) that was accompanied by a fluctuation of TLR4 protein levels (**Fig. 4C**). Among the CRF_2_ agonists, the suppressive effect of UCN2 was the most prominent. The CRF_1_ agonists were ineffective in altering TLR4 expression and the production of interleukins on pre-adipocytes (data not shown).

Exposure of pre-adipocytes to LPS did not alter the rates of IL-6 and CXCL1 production compared to parallel control cells that were not exposed to LPS (**Fig. S2A**). At 3 hr exposure to LPS, a transient suppression of TLR4 mRNA expression was observed which returned to basal levels at 12 hrs (**Fig. S2B**). This transient suppression of TLR4 mRNA expression was not accompanied by a significant alteration in the expression of TLR4 protein as measured by Flow Cytometry (**Fig. S2C**).

### Effect of UCN2 but not of LPS on Adiponectin Production by Pre-adipocytes

Short term exposure (3 to 10 hrs) of pre-adipocytes to UCN2 was ineffective in altering their production of adiponectin. However, longer exposures (24 to 48 hrs) of pre-adipocytes to UCN2 suppressed the production of adiponectin. More specifically, a 48-hr exposure of pre-adipocytes to UCN2 suppressed production of adiponectin by 49.4±1.6% (*p<0.001*) compared to parallel controls (100±0.5%). All other CRF agonists provoked a borderline (but not statistically significant) suppression of adiponectin production, while exposure of pre-adipocytes to LPS was ineffective in altering adiponectin production compared to parallel adipocytes not exposed to LPS (data not shown). Leptin was undetectable in pre-adipocytes even following exposure to LPS (data not shown).

### Effect of LPS on the Production of Inflammatory Adipokines and the Expression of TLR4 in Differentiated Adipocytes

**Figure 5:**
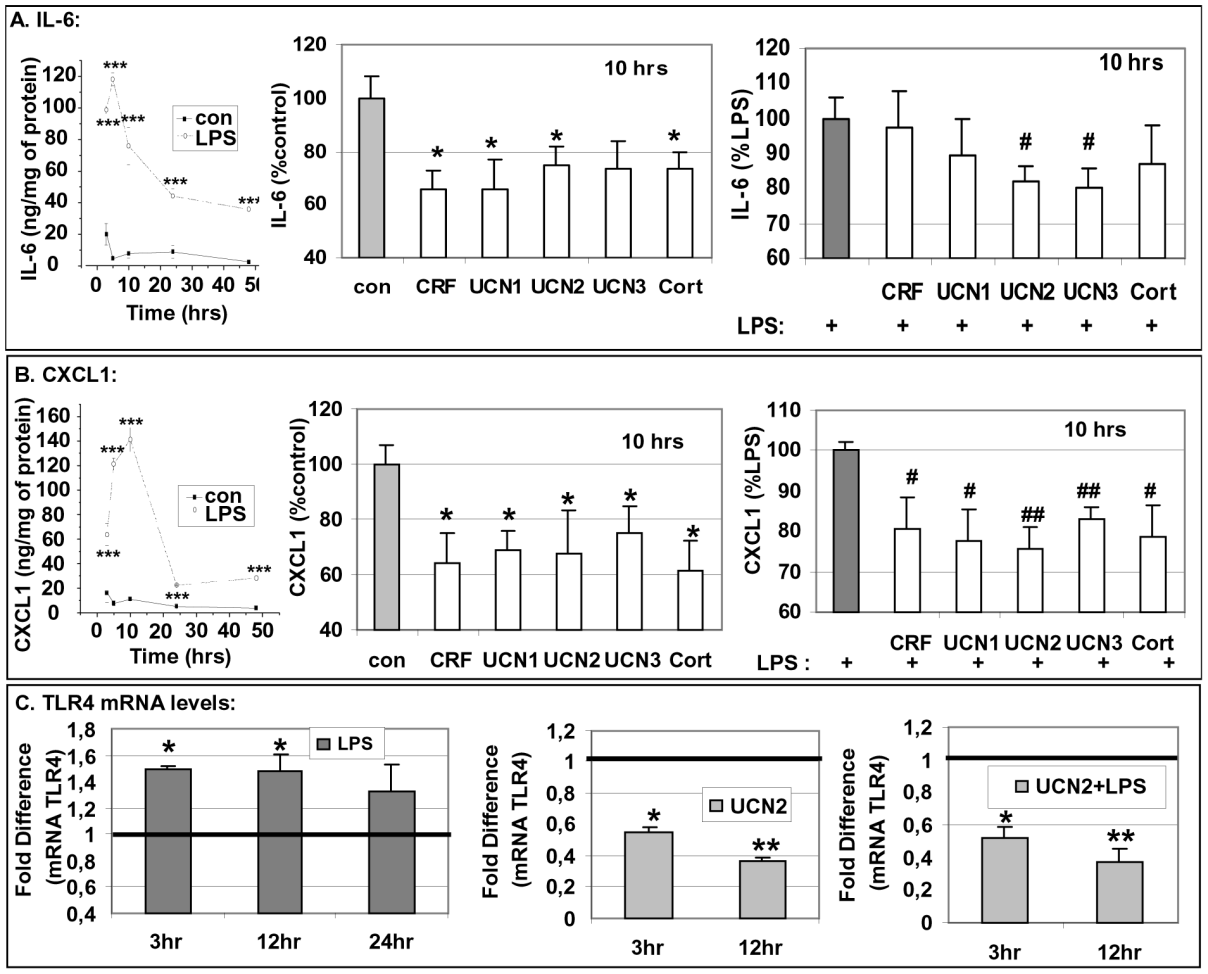
CRF agonists and LPS differentially regulate TLR4 expression and interleukins production by mouse mature adipocytes. Mature adipocytes were exposed to CRF peptides (CRF, UCN1, UCN2, UCN3, Cortagine) all at 10^−8^M plus/minus LPS (10 ng/ml) and the production of IL-6 (Panel A), CXCL1 (Panel B) was measured. Data are expressed as mean±SE, n = 10 of five independent experiments. *p<0.05, **p<0.01 and ***p<0.001 depict the statistical significant difference from cells exposed only to vehicles, while ^#^p<0.05, ^##^p<0.01 depict the statistical significant difference from cells exposed to LPS alone.

**Figure 6:**
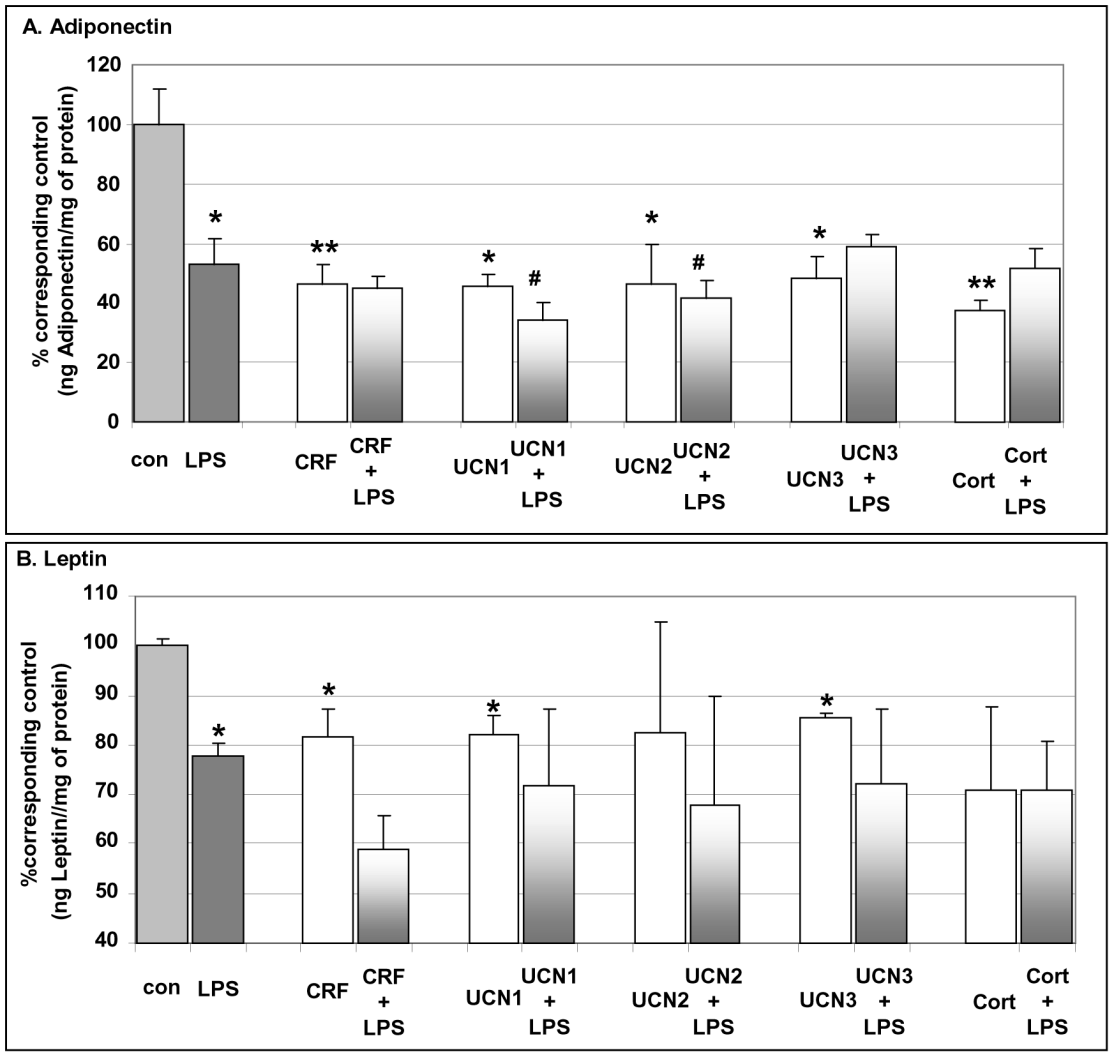
CRF agonists and LPS affect the release of adipokines by differentiating adipocytes. Differentiating adipocytes were exposed to CRF peptides (CRF, UCN1, UCN2, UCN3, Cortagine) all at 10^−8^ M plus/minus LPS (10 ng/ml) and the release of adiponectin (Panel A) or leptin (Panel B) was measured. Data are expressed as mean±SE, n = 10 of five independent experiments. *p<0.05, **p<0.01 depict the statistical significant difference from cells exposed only to vehicles, while ^#^p<0.05 depicts the statistical significant difference from cells exposed to LPS alone.

To examine the effect of LPS on the production of IL-6, CXCL1, adiponectin, leptin and on the expression of TLR4 receptor in fully differentiated adipocytes, we exposed them to LPS for different time intervals. Fully differentiated adipocytes exposed to LPS exhibited a remarkable increase in IL-6 and CXCL1 production (**Fig. 5A, B** **, left graphs**) while at the same time (at 5 hrs) the production of adiponectin and leptin was suppressed (**Fig. 6**). The peak effect of LPS on IL-6, CXCL1, adiponectin and leptin production by the differentiated adipocytes took place between 5 to 10 hrs of exposure. More specifically, exposure to LPS for 5 hrs had the following effects: (a) it increased the production of IL-6 to 118.0±4.1 (ng IL-6/mg of protein, *p<0.001*) compared to 4.6±0.4 ng IL-6/mg of protein of control cells (parallel cells not exposed to LPS) (**Fig. 5A** **, left graph**), (b) LPS induced the production of CXCL1 to 121.3±4.8 (ng CXCL1/mg of protein, *p<0.001*) compared to 7.5±2.1 ng CXCL1/mg of protein of control cells (**Fig. 5B** **, left graph**), and (c) LPS suppressed the production of adiponectin and leptin (**Fig. 6**). Furthermore, a prolonged increase of TLR4 mRNA levels was evident between 3 to 24 hrs of exposure to LPS of differentiated adipocytes compared to parallel basal TLR4 mRNA levels in unexposed cells (**Fig. 5C** **, left graph**).

### Effect of CRF Peptides on the Production of Inflammatory Adipokines and TLR4 Expression in Differentiated Adipocytes

To examine the effect of CRF agonists on the production of interleukins by fully differentiated adipocytes, we exposed them to CRF agonists for a variety of time intervals and measured IL-6, CXCL1, TLR4 mRNA and the adipokines adiponectin and leptin. The resulting data are depicted in **Figure 5** **(panels A & B, middle graphs)**. All CRF receptor agonists were effective in attenuating the inflammatory profile of fully differentiated adipocytes. More specifically, cells exposed to CRF, UCN1 (agonists of both CRF_1_ and CRF_2_ receptors), UCN2, UCN3 (agonists of CRF_2_ receptor) or Cortagine (a CRF_1_ receptor agonist) for 10 h suppressed: (a) IL-6 secretion to 65±6% (*p<0.05*), 65±11% (*p<0.05*), 74±7% (*p<0.05*), 73±10% or 73±6% (*p<0.05*), respectively compared to IL-6 from parallel control cells (100±8%) (**Fig. 5A** **, middle graph**); (b) CXCL1 production was suppressed to 64±11% (*p<0.05*), 69±6% (*p<0.05*), 67±15% (*p<0.05*), 74±10% (*p<0.05*) or 61±10% (*p<0.05*), respectively compared to the parallel control cells (100±6%) (**Fig. 5B** **, middle graph**).

Furthermore, UCN2 suppressed TLR4 mRNA expression in differentiated adipocytes compared to parallel control adipocytes (**Fig. 5C** **, middle graph**).

CRF peptides also suppressed basal production of adiponectin and leptin by differentiated adipocytes. Indeed, exposure to CRF, UCN1, UCN2, UCN3 or Cortagine suppressed: (a) adiponectin to 46±6% (*p<0.01*), 45±3% (*p<0.05*), 46±13% (*p<0.05*), 48±7% (*p<0.05*) or 37±3% (*p<0.01*), respectively compared to parallel control cells (100±12%) (**Fig. 6A**); (b) Leptin was suppressed to 81±5% (*p<0.05*), 82±3% (*p<0.05*), 82±22%, 85±0.5% (*p<0.05*) or 70±16%, respectively compared to parallel control cells (100±1%) (**Fig. 6B**).

### Effect of CRF Peptides on the LPS-induced Production of Inflammatory Cytokines and TLR4 Expression in Differentiated Adipocytes

As shown above, LPS was a potent inducer of inflammation in differentiated adipocytes. To determine the effect of CRF agonists on LPS-induced interleukins production in fully differentiated adipocytes, we exposed them to various CRF agonists in the presence or absence of LPS for a variety of time intervals and measured IL-6 and CXCL1. The results showed that only the CRF_2_ receptor agonists were effective in attenuating LPS-induced IL-6 secretion by differentiated adipocytes, while all CRF receptor agonists were effective in attenuating LPS-induced CXCL1 secretion by differentiated adipocytes. More specifically, exposure of adipocytes to UCN2 or UCN3 for 10 h suppressed LPS-induced IL-6 production to 81±4% (*p<0.05*) or 80±5% (*p<0.05*), respectively as compared to IL-6 produced from cells exposed to LPS alone (100±5%) (**Fig. 5A** **, right graph**). Adipocytes exposed to CRF, UCN1, UCN2, UCN3 or Cortagine for 10 h resulted in suppression of CXCL1 production to 80±7% (*p<0.05*), 77±8% (*p<0.05*), 75±5% (*p<0.01*), 83±2% (*p<0.01*) or 78±7% (*p<0.05*), respectively as compared to CXCL1 produced from cells exposed to LPS alone (100±2%) (**Fig. 5B** **, right graph**). Regarding expression of TLR4, the results showed that LPS was a potent inducer of its mRNA expression and UCN2 significantly suppressed the effect of LPS (**Fig. 5C** **, right graph**).

### Effect of CRF Peptides on the LPS-mediated Adipokines Production by Differentiated Adipocytes

Data above indicated that exposure to LPS suppressed adiponectin and leptin production by differentiated adipocytes. To determine the effect of CRF agonists on LPS-mediated suppression of adiponectin and leptin production by differentiated adipocytes, we exposed them to CRF peptides in the presence or absence of LPS for different time intervals and measured both adipokines. We found that only the CRF_2_ receptor agonists were effective in enhancing LPS-mediated suppression of adiponectin secretion from differentiated adipocytes, while they were not able to alter LPS-mediated suppression of leptin production from differentiated adipocytes. More specifically, adipocytes exposed to UCN1 or UCN2 for 5 h enhanced adiponectin suppression to 65±10% (*p<0.05*) or 78±11% (*p<0.05*), respectively compared to parallel adipocytes exposed to LPS alone (100±2%) (**Fig. 6A**).

### Effect of LPS in the Metabolic and Inflammatory Phenotype of Human Mature White Adipocytes

**Figure 7:**
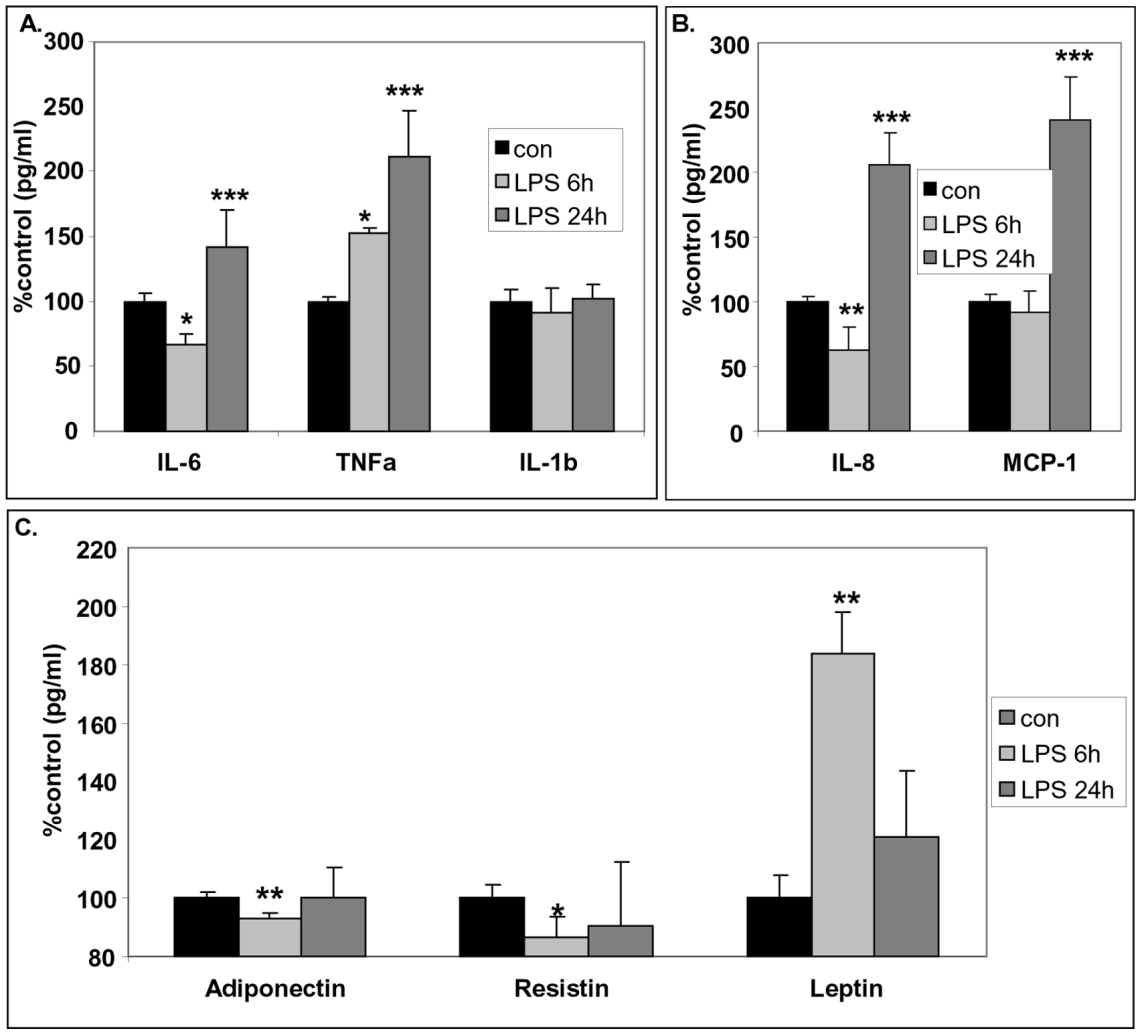
Time-dependent effect of LPS in the metabolic and inflammatory components of human white adipocytes. Cells were exposed to 10/ml LPS for 6h or 24 h and the release of IL-6, TNF-α, IL-1b (Panel A), IL-8, MCP-1 (Panel B), adiponectin, resistin, leptin (Panel C) were measured. Data are expressed as mean±SE, n = 6 of three independent experiments. *p<0.05, **p<0.01 and ***p<0.001 depict the statistical significant difference from cells exposed only to vehicles.

LPS was a powerful inducer of pro-inflammatory cytokines and chemokines in human mature white adipocytes. Indeed, 6-hr incubation of white adipocytes with LPS (10 ng/ml) transiently reduced IL-6 and IL-8 levels, while 24-hr incubation with LPS up-regulated the release of IL-6 and IL-8. The production of TNF-α was progressively induced remaining at high levels after 24-hr exposure to LPS, while the production of MCP-1 was acutely increased. It is interesting that 6-hr exposure to LPS down-regulated adiponectin and resistin levels, while leptin levels were highly up-regulated. Those statistical significant effects were transient, as there were lost in the 24-hr exposure to LPS for adiponectin, resistin and leptin (**Fig. 7**).

### Effect of CRF and UCN1 in the Inflammatory Phenotype of Human Mature White Adipocytes

**Figure 8:**
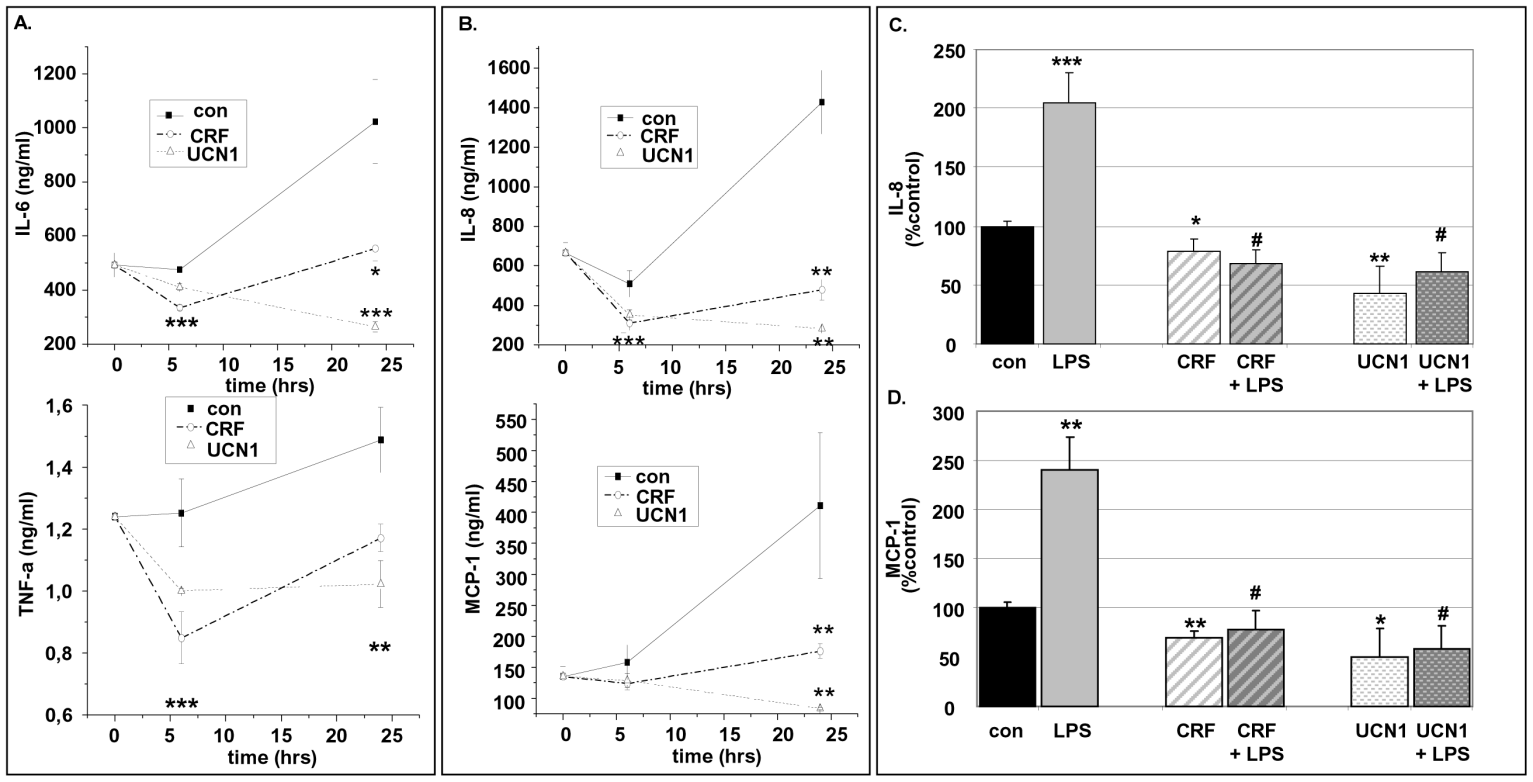
CRF and UCN1 down-regulate the basal production of interleukins and chemokines and the LPS-induced pro-inflammatory effect in human white adipocytes. Cells were exposed to CRF or UCN1 both at (10^−8^ M) for different time intervals and the release of IL-6, TNF-α (Panel A), IL-8 and MCP-1 (Panel B) was measured. Panel C, Human white adipocytes were exposed to CRF, UCN1 both at (10^−8^ M) and/or LPS (10 ng/ml) for 24 hrs and the production of IL-8 and MCP-1 was measured. Data are expressed as mean±SE, n = 6 of three independent experiments. *p<0.05, **p<0.01 and ***p<0.001 depict the statistical significant difference from cells exposed only to vehicles, while ^#^p<0.05 depicts the statistical significant difference from cells exposed to LPS alone.

CRF and UCN1 were powerful regulators of basal interleukins and chemokines levels in human white adipocytes. Indeed, 6-hr exposure of human white adipocytes to CRF or UCN1 both at (10^−8^ M) managed to decreased the levels of IL-6, TNF-α, IL-8 and MCP-1 compared to control cells (e.g. cells exposed only to vehicles). These statistical significant effects were still visible at 24-hr of exposure to CRF or UCN1 (**Fig. 8A, B**).

### CRF and UCN1 were Potent Regulators of LPS-induced Pro-inflammatory Effect in Human Mature White Adipocytes

In this set of experiments we measured the production of chemokines IL-8 and MCP-1 from human white adipocytes exposed to the CRF or UCN1 in the presence or absence of LPS for 24 hrs. Exposure to LPS provoked a spectacular increase in IL-8 and MCP-1 production. Exposure of adipocytes to CRF or UCN1 both at (10^−8^ M) suppressed the LPS-induced IL-8 and MCP-1 production (**Fig. 8C**).

## Discussion

CRF and its related peptides, the urocortins, have a major role in triggering and maintaining the homeostatic response both systematically and locally, in a paracrine mode of action. We and others have shown that the CRF peptides appear to play a key role in the regulation of the inflammatory response at the innate immunity level and more specifically on macrophage differentiation and function [1]–[4]. The aim of the present work was to examine the role of the CRF family of neuropeptides on the immune phenotype of adipocytes. It should be noted here that obesity induces a pro-inflammatory phenotype of visceral adipocytes resulting in the development of generalized chronic low grade inflammation, the basis of insulin resistance, diabetes mellitus and cardiovascular disease. Adipocytes share multiple phenotypical features of macrophages and within the obese adipose tissue they cross-talk causing a spiralling intra-abdominal and later generalized inflammation [17]. We have found that both CRF receptors and CRF-related peptides are expressed in human visceral white adipocytes, mouse 3T3L1 pre-adipocytes and in their *in vitro* differentiation into mature, lipid-containing adipocytes. We have also found that the CRF_1_ and CRF_2_ agonists suppress their differentiation and basal and LPS-induced inflammatory profile. CRF_2_ agonists suppressed mainly the inflammatory response while CRF_1_ agonists suppressed their differentiation to mature adipocytes.

Adipocytes and macrophages share common characteristics including the ability to recognise microbial components like LPS by the cell surface TLR receptors which activate the NFkB pathway that leads to the production of cytokines and the presence of TNF-α receptors on their surface [10]. This capability of adipocytes is of paramount importance in obesity, because it has been suggested that in obese individuals LPS from gram-negative bacteria is up-regulated further inducing the development of the generalized low grade inflammation of obesity [18]. In addition TLR4 signals can be triggered by apoptotic cell debris that bind on the receptor and are abundant in the obese adipose tissue because of the extensive adipocytes apoptosis due to the well-documented local hypoxia. In this environment, adipocytes like monocytes and macrophages develop phagocytic capabilities towards the apoptotic cells due to hypoxia [13], expressing macrophage-specific antigens in an environment rich in macrophages [19].

Our study indicates that the 3T3L1 mouse pre-adipocytes as well as *in vitro* differentiated mature adipocytes expressed the TLR4 receptor and produced IL-6 and CXCL1. Moreover, prolonged exposure of pre-adipocytes to LPS, in conditions favouring differentiation to mature adipocytes, accelerated their differentiation rate. More specifically, LPS induced the formation of lipid droplets in the cells (a central marker of adipocyte maturation), the expression of receptor TLR4 (regulating activation of the NFkB pathway in macrophages), the production of interleukins (the final products of the NFkB pathway), and suppressed the production of the anti-inflammatory adipokine, adiponectin.

Furthermore, in fully differentiated adipocytes, exposure to LPS provoked a significant and sustained increase in the expression of TLR4 as well as the production of IL-6 and CXCL1 and a significant decrease to almost half of adiponectin and leptin. Our data are in agreement with previously published findings illustrating that LPS provokes an increase (800%) in IL-6 secretion and a decrease (30%) in leptin production by adipocytes [20]. Moreover, the fact that adiponectin was also down-regulated by LPS could be associated with induction of IL-6, which has been reported to negatively regulate adiponectin secretion in 3T3L1 cells [21].

In our hands, pre-adipocytes and mature adipocytes produced IL-6 in comparable amounts. Furthermore, we have found that short exposure of pre-adipocytes to LPS did not affect their basal inflammatory phenotype (measuring the levels of IL-6, CXCL1 and TLR4) or the levels of adiponectin produced. Interestingly, LPS dramatically increased the production of IL-6 and CXCL1 in fully differentiated adipocytes. It should be noted that both populations express the TLR4. These data suggest that mature adipocytes are more vulnerable to develop a pro-inflammatory phenotype than pre-adipocytes. Our data are in agreement with the findings demonstrating that the increase in number and size of hyperplastic adipocytes of obese contribute to the maintenance of low-grade inflammatory state in obesity [22]; [23]. In contrast to our results, a study by Harkins and colleagues demonstrated that 3T3L1 pre-adipocytes secret higher levels of IL-6 than differentiated adipocytes. The discrepancy from our data might lie on the fact that the authors incubated pre-adipocytes with differentiating medium for only 3 days and the cells might have not fully differentiated.

We also examined the role of CRF_1_ and CRF_2_ agonists on the inflammatory phenotype of pre-adipocytes as well as of mature adipocytes. There was only a transient suppressive effect in basal interleukins production and TLR4 expression after exposure of pre-adipocytes to the CRF_2_ agonists. In contrast, differentiated adipocytes responded rapidly to these agents altering their inflammatory phenotype. Indeed, there was a substantial pro-inflammatory effect of LPS in differentiated adipocytes and a shorter in magnitude but significant anti-inflammatory effect of both CRF_1_ and CRF_2_ agonists. We conclude that the phenotype of differentiated adipocytes is more pro-inflammatory compared to pre-adipocytes. Indeed, mature adipocytes can be triggered very quickly to up-regulate or minimize the production of inflammatory molecules in response to the appropriate stimulus.

The expression of the CRF family of neuropeptides and their receptors are widely distributed throughout the body. CRF_1_ receptor is predominantly expressed in the brain while CRF_2_ in mainly expressed in the periphery [24]. We have found that the differentiated adipocytes have a significant higher number of CRF_2_ binding sites compared to mouse pre-adipocytes. The presence of a complete CRF system within adipose tissue has already been published for humans [7]. Interestingly, it was proposed that the expression of the receptor CRF_1_ is stronger in human subcutaneous fat, while the expression of the receptor CRF_2_ in human visceral fat. The inflammatory identity of the adipose tissue seems to depend to the origin of the fat depot. Indeed, visceral fat is believed to be related to a pro-inflammatory phenotype linking to metabolic and cardiovascular problems in contrast to the subcutaneous fat [25]. In line to this finding, we propose that the anti-inflammatory effect of CRF peptides in differentiated adipocytes was via CRF_2_ that seems to be highly up-regulated in these pro-inflammatory cells compared to pre-adipocytes.

CRF receptors in adipocytes appear to play a role on their differentiation and on the development of a pro-inflammatory phenotype. More specifically, CRF_1_ agonists suppressed the differentiation rate of pre-adipocytes to mature adipocytes, an important step towards the development of obesity. On the other hand, CRF_2_ agonists transiently suppressed the development of a pro-inflammatory phenotype of pre-adipocytes under basal conditions, while exposure to either CRF_1_ or CRF_2_ agonists suppressed the development of a pro-inflammatory phenotype of mature adipocytes under basal and LPS-induced inflammation. The pro-inflammatory phenotype of adipocytes was assessed by the expression of TLR4 and the production of IL-6 and CXCL1.

The receptor TLR4 is a trans-membrane protein that is highly expressed in cells of the innate immune system as well as other cell types like adipocytes. Vitseva and colleagues have shown that the expression of TLR4 is up-regulated in the adipose tissue and is associated with the activation of the NFkB pathway [26]. Moreover, experiments in mice with a mutation in the receptor TLR4 seem to protect these mice against the development of diet-induced obesity [27]. In obesity, the major source of circulating IL-6 is the adipocyte as well as adipose tissue macrophages [28]. The induction of the circulating levels of IL-6 is paramount for the activation of the mechanisms for innate immune response. The suppression of basal and LPS-induced expression of TLR4 receptor and the inhibition of the production of interleukins by the CRF neuropeptides could be a major pathway in containing obesity-induced chronic low grade inflammation.

Previously published studies from our lab have documented both pro- and anti-inflammatory effects of the CRF family of peptides at the level of macrophages. Indeed, in RAW264.7 macrophages, CRF agonists augmented the pro-inflammatory effect of LPS by inducing TLR4 gene expression, through CRF_2_, via activation of the transcription factors PU.1 and AP-1 [1]. However, CRF_1_ and CRF_2_ agonists exert an anti-inflammatory effect on Kupffer cells (the resident macrophages in the liver) by inhibiting the LPS-triggered TNF-α production [3]. CRF_1_ and CRF_2_ agonists also exert an anti-inflammatory effect in primary mouse macrophages during the early phase of inflammation suppressing LPS-induced TNF-α release from macrophages via induction of COX-2 and PGE2 [2], and, endogenous CRF_2_ agonists exert anti-inflammatory effects in macrophages by inducing their apoptosis [4]. Our current data support our previous findings suggesting that the CRF system within adipose tissue may play a regulatory role on metabolic inflammation in obesity.

CRF_2_ agonists were more potent than CRF_1_ agonists in reducing the adiponectin secretion in both pre-adipocytes and differentiated white adipocytes. It should be noted that leptin secretion was undetectable in pre-adipocytes, in contrast to fully differentiated adipocytes as recently published [20]. Furthermore, CRF agonists were potent in reducing the basal leptin secretion in fully differentiated adipocytes. These data are in agreement with the hypothesis that the induction of leptin production in obesity is a mechanism to contain further accumulation of fat on adipose tissue. Indeed, leptin stimulates the anorexiogenic part of the arcuate nucleus in the hypothalamus and at the same time it suppresses its orexiogenic part. It also stimulates resting energy expenditure [29]. These effects are mediated via the POMC and CRF neuropeptides within the central nervous system. In the present study, we propose that the induction of the CRF system within adipose tissue could serve as a homeostatic mechanism in order to ameliorate the deleterious effects of triglyceride accumulation within adipocytes by directly reducing leptin levels.

Our data are in agreement with previously published findings indicating that a glycerol-3-phosphate dehydrogenase activity (a marker of adipocytes differentiation) was positively correlated with leptin release [30]. It should be noted that freshly isolated human pre-adipocytes do not exhibit significant leptin mRNA and protein levels, while during differentiation leptin mRNA and protein levels were increased in accordance with the cellular lipid accumulation [31]. It has been suggested that the levels of leptin mRNA increased several-fold in parallel to typical adipocyte markers like lipoprotein lipase, adipsin and glycerophosphate dehydrogenase during the differentiation of pre-adipocytes 3T3L1 to mature adipocytes [32].

CRF_1_ agonists suppressed the differentiation process of pre-adipocytes to mature adipocytes that was followed by a parallel suppression of both IL-6 and leptin production. Indeed, like leptin [33], IL-6 is an anorexiogenic factor that act centrally to reduce food intake and increase body expenditure [34]–[36]. We postulate that the suppressing effect of CRF neuropeptides on IL-6 production from adipocytes may partially explain the post-stress orexiogenic period.

### In conclusion

We have found that both CRF receptors are expressed in mouse 3T3L1 pre-adipocytes and in their *in vitro* differentiation into mature, lipid-containing adipocytes as well as in human visceral adipocytes. We have also found that the CRF_1_ and CRF_2_ agonists suppressed their differentiation and basal and LPS-induced inflammatory profile. More specifically, CRF_2_ agonists mainly suppressed the inflammatory response while CRF_1_ agonists suppressed their differentiation to mature adipocytes.

## Supporting Information

Figure S1 **Effect of Cortagine and LPS on interleukins and adipokines during differentiation of 3T3L1.** Pre-adipocytes were cultured in differentiating media supplemented with Cortagine at 10^−8^ M plus/minus LPS (10 ng/ml) and the production of interleukins and adipokines was measured by ELISA. Data are expressed as mean±SE, n = 10 of five independent experiments. *p<0.05, **p<0.01, ***p<0.001 depict the statistical significant difference from cells exposed only to vehicles, while ^#^p<0.05 and ^##^p<0.01 depict the statistical significant difference from cells exposed to LPS alone.
(TIF)

Figure S2 **LPS is ineffective in interleukin and TLR4 production by pre-adipocytes.** Pre-adipocytes were exposed to LPS at 10 ng/ml and the production of interleukins was measured by ELISA (Panel A), the TLR4 mRNA levels were measured by RT-PCR (Panel B) and TLR4 protein levels were measured by FACS analysis (Panel C) at several time intervals. Data are expressed as mean±SE, n = 6 of three independent experiments.
(TIF)
