# Supplementary figures and images for: Corticotrophin-Releasing Factor (CRF) and the Urocortins Are Potent Regulators of the Inflammatory Phenotype of Human and Mouse White Adipocytes and the Differentiation of Mouse 3T3L1 Pre-Adipocytes
Source: PLoS One. 2014 May 16;9(5):e97060. doi: 10.1371/journal.pone.0097060 (PMC4024041; doi:10.1371/journal.pone.0097060)

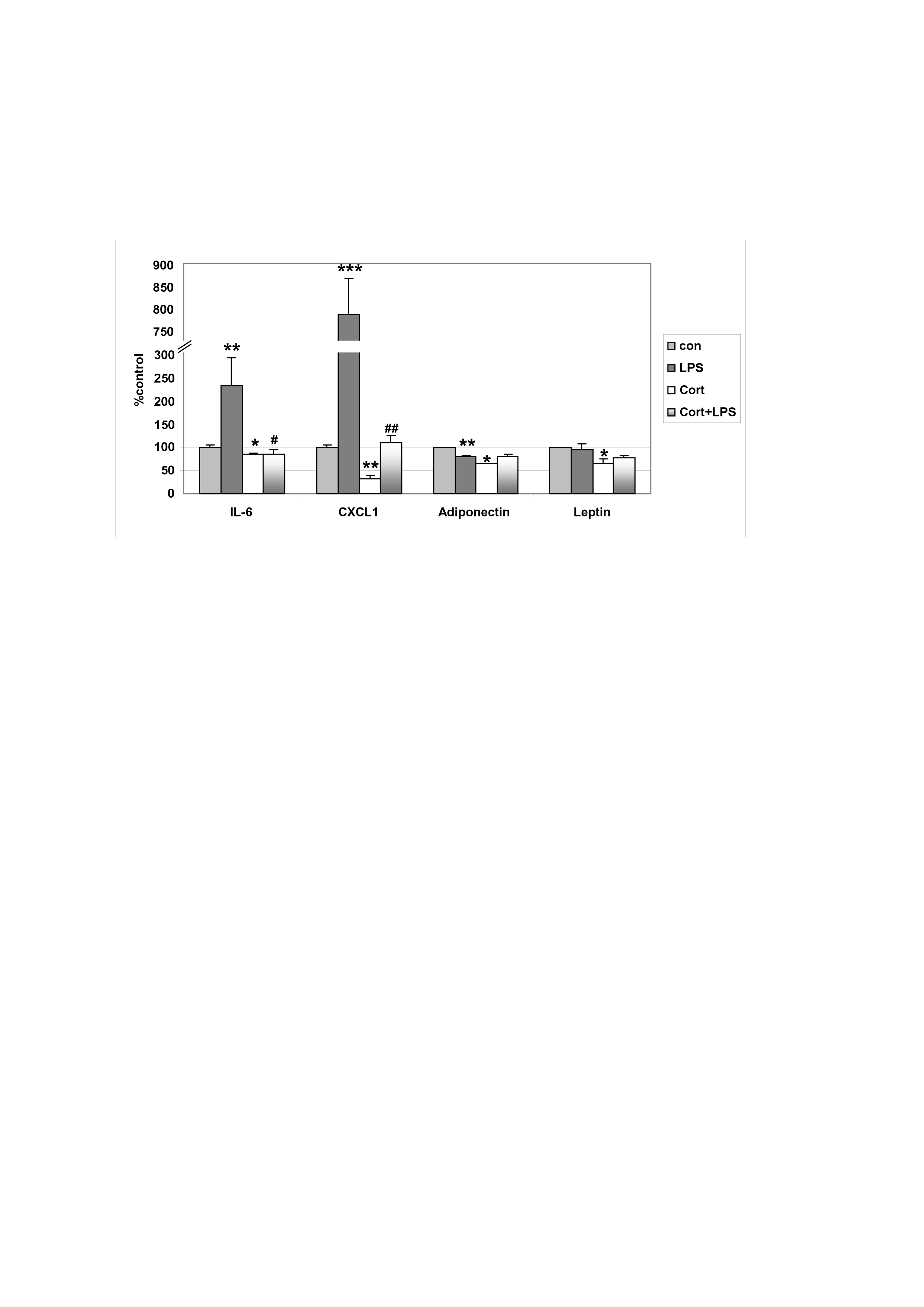

Supplement: Figure S1 — Effect of Cortagine and LPS on interleukins and adipokines during differentiation of 3T3L1. Pre-adipocytes were cultured in differentiating media supplemented with Cortagine at 10−8 M plus/minus LPS (10 ng/ml) and the production of interleukins and adipokines was measured by ELISA. Data are expressed as mean±SE, n = 10 of five independent experiments. *p<0.05, **p<0.01, ***p<0.001 depict the statistical significant difference from cells exposed only to vehicles, while #p<0.05 and ##p<0.01 depict the statistical significant difference from cells exposed to LPS alone. (TIF) [file pone.0097060.s001.tif]

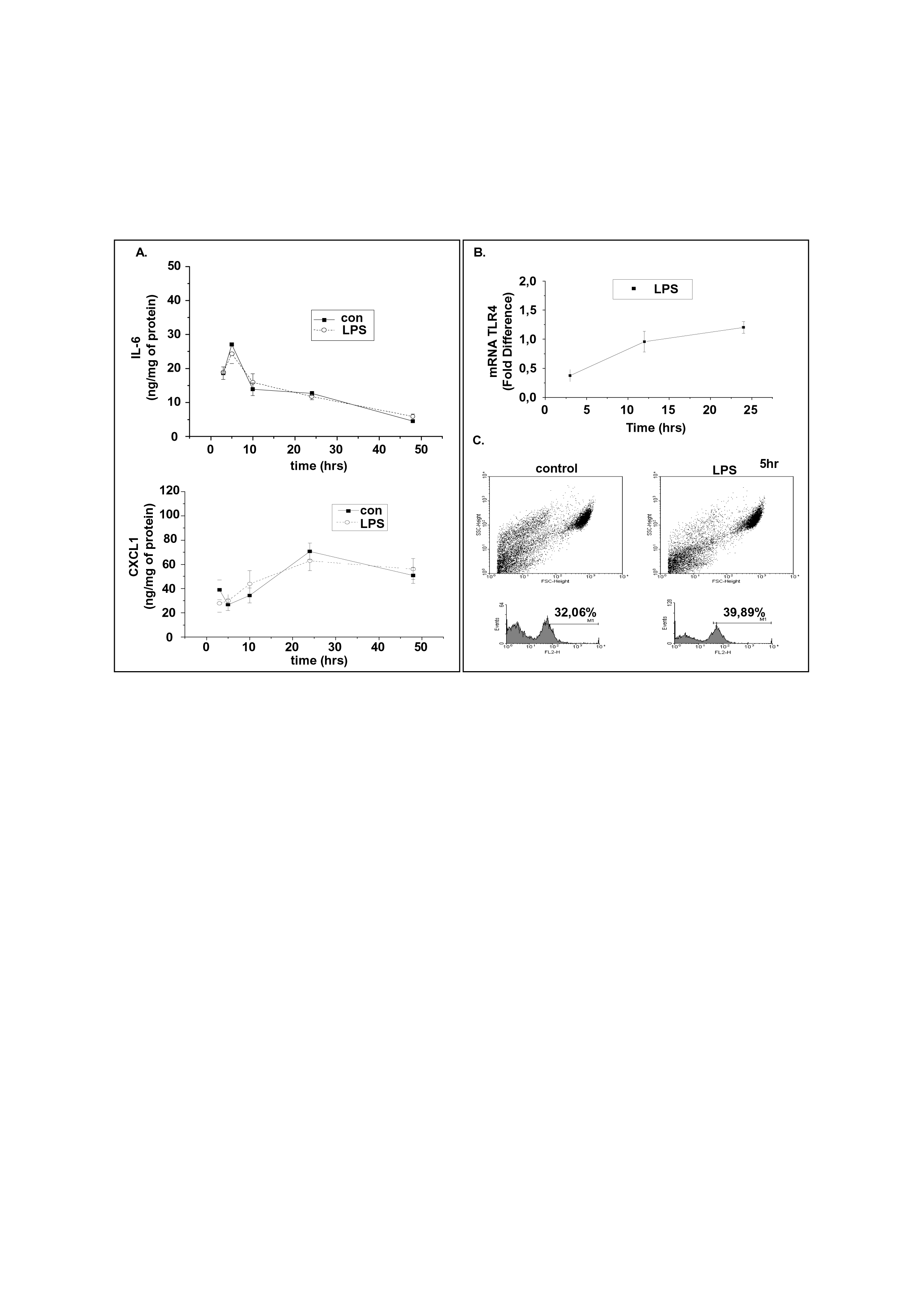

Supplement: Figure S2 — LPS is ineffective in interleukin and TLR4 production by pre-adipocytes. Pre-adipocytes were exposed to LPS at 10 ng/ml and the production of interleukins was measured by ELISA (Panel A), the TLR4 mRNA levels were measured by RT-PCR (Panel B) and TLR4 protein levels were measured by FACS analysis (Panel C) at several time intervals. Data are expressed as mean±SE, n = 6 of three independent experiments. (TIF) [file pone.0097060.s002.tif]
